# Factors for the integration of prevention in primary care: an overview of reviews

**DOI:** 10.3399/BJGPO.2023.0141

**Published:** 2024-08-21

**Authors:** Estelle Clet, Pierre Leblanc, François Alla, Christine Cohidon

**Affiliations:** 1 Prevention Department, University Hospital Centre Bordeaux Division of Public Health, Bordeaux, France; 2 I-prev/PHARES (INSERM U1219), Université de Bordeaux, Bordeaux, France; 3 Institute of Public Health Epidemiology and Development, Prevention Research Chair Bordeaux, Bordeaux, France; 4 Quality and Population Health Department, Civil Hospices of Lyon, Lyon, France; 5 Research On Healthcare Performance (RESHAPE), Claude Bernard Lyon 1 University (INSERM U1290), Lyon, France; 6 Department of Family Medicine, Center for Primary Care and Public Health (Unisanté), University of Lausanne, Lausanne, Switzerland

**Keywords:** prevention, preventive health services, primary health care, service organisation, general practice

## Abstract

**Background:**

The global burden of non-communicable diseases is increasing and the need for prevention is huge. Policies have yet to produce results and prevention indicators remain low. Primary care (PC) represents an opportunity to optimise the practice of prevention, but GPs are coming up against barriers that are holding back their prevention practices.

**Aim:**

To identify the barriers and facilitators for the implementation of routine prevention practices in PC.

**Design & setting:**

This study is an international overview of reviews focusing on the integration of prevention in PC settings.

**Method:**

The search was conducted in July 2022 using MEDLINE, Embase, Web of Science, and the Cochrane Database of Systematic Reviews. Included reviews are systematic reviews or scoping reviews adopting a systematic approach.

**Results:**

The 35 reviews included identify multiple barriers and facilitators related to the integration of prevention in PC. These factors are heterogeneous with regard to their source (the patient, the professional, and the health system) and their level of action (individual, organisational, or contextual). The results show the need to organise PC at the professional level (for example, in training), at the local level (for example, the information system), and at the political level (for example, the unclear definition of the role of professionals).

**Conclusion:**

The factors influencing the integration of prevention in PC are multiple and act at different levels (individual, organisational, and health-system level). Organisation factors play a major role and seem to be a means of overcoming the difficulties encountered by healthcare professionals in developing preventive practices.

## How this fits in

Many factors influence the practice of prevention in primary care. Many of these have already been identified, but the organisational aspect has so far been little explored in this context. In view of the current changes and structuring of primary care in many countries, the results of this overview of reviews could help health professionals and health authorities to integrate prevention into these structural changes.

## Introduction

Non-communicable diseases (for example, cardiovascular diseases, diabetes, chronic respiratory diseases) are responsible for 74% of deaths worldwide.^1^ These deaths are partly preventable through a reduction in behavioural risk factors such as tobacco and alcohol consumption, diet, and physical activity.^1^ To act on these risk factors, primary care (PC) providers are indispensable. They have regular contact with a large section of the population and can encourage early attention to health.^2^ Prevention and health promotion services are also an integral part of the PC mission, as defined by the World Health Organization (WHO) at the international conference on PC in Alma Ata in 1978.^3^

Accordingly, prevention in PC is the subject of several health policy strategies in various countries.^4,5^ However, these policies have yet to produce significant results in the field and prevention indicators remain low. In 2019, almost 60% of adults in Organisation for Economic Co-operation and Development (OECD) countries were overweight or obese.^6^ PC professionals, particularly GPs, are aware of the value of prevention and are motivated to promote this approach.^7–10^ However, they face many obstacles that prevent them from systematising prevention approaches,^11,12^ making the health outcomes still heterogeneous. For example, worldwide, 59% of women have a diagnosis of hypertension; of these 47% are treated, and only 23% have controlled hypertension.^13^

To improve the integration of prevention into the practices of PC professionals, it is necessary to describe in detail the context in which they operate. Many factors (barriers and facilitators) have already been identified in other settings^14^ or with a focus on behavioural change.^15^ The organisational aspect appears to be an important lever in PC professionals to integrate prevention into their practices.^14^ However, to the best of our knowledge, no systematic synthesis has yet focused on the organisation aspect of prevention practice in PC. This approach makes it possible to produce an overall view of the factors influencing the practice of prevention in PC. The aim of this overview of reviews is therefore to identify the barriers and facilitators for the implementation of prevention practice in PC.

## Method

This is an overview of reviews. It is a systematic review of systematic reviews; that is, it includes any kind of literature review with a rigorous methodology to achieve a single synthesis of a specific topic.^16^ This overview of reviews was conducted in accordance with the recommendations of the Preferred Reporting Items for Systematic Reviews and Meta-Analyses (PRISMA) statement^17^ and guidance on performing mixed-methods systematic reviews.^18^

### Data searches

The search equation was developed with a librarian from the University of Bordeaux and includes the keywords 'prevention', 'primary care', 'barriers' OR 'facilitators', and their synonyms. The search was conducted on 5 July 2022 in MEDLINE, Embase, Web of Science, and the Cochrane Database of Systematic Reviews (Supplementary Box 1). No date or location restrictions were applied. Only articles published in English were included. The data were managed on the Covidence platform.

### Study selection

This study focuses on the integration of prevention of risk factors in the routine practices of PC providers. The following two types of preventive intervention were included: (i) primary prevention interventions, which aim to reduce the incidence of chronic conditions in the general population (for example, vaccination); and (ii) secondary prevention, which aims to detect chronic conditions early in a population sample with risk factors (for example, screening). Reviews addressing only tertiary and quaternary prevention have been excluded. We have also included articles on prevention in general without a specific theme.

In this overview of reviews, the PC setting follows the WHO definition.^19^ Therefore, reviews dealing with emergency departments or hospitals have not been included. As concerns the targeted population, all patients in PC were included (adults and older people) excluding children and adolescents, as well as specific communities (for example, migrants, disabled people), owing to their particular care pathways. We have included systematic reviews as well as scoping reviews with a systematic approach. Qualitative and quantitative reviews are both included. All inclusion and exclusion criteria are presented in Supplementary Table S1.

Titles and abstracts were independently and blindly reviewed by two reviewers. Conflicting abstracts were resolved by reading the full text. Eligible full texts were read independently by two reviewers to be included in the final study. Conflicts were resolved through discussion or by the involvement of a third reviewer if no consensus could be reached.

### Data extraction

A data extraction form was designed specifically for this study. It contained the following information: identification of the review (title, authors, and date); objectives; prevention theme; search and analysis method; number of primary articles included; outcomes (barriers and facilitators); and the risk of bias assessment. Data extraction was carried out by two reviewers.

### Quality assessment

For all reviews included in the study, a reviewer assessed the methodological quality using the ROBIS tool.^20^ A quality score was assigned as follows: high risk of bias; low risk of bias; and unclear risk of bias.

### Data synthesis and analysis

The data synthesis followed a convergent integrated approach.^18^ The first step was to translate the quantitative data into qualitative data by means of a textual description. Then the two types of data were put together.

**Figure 1.**
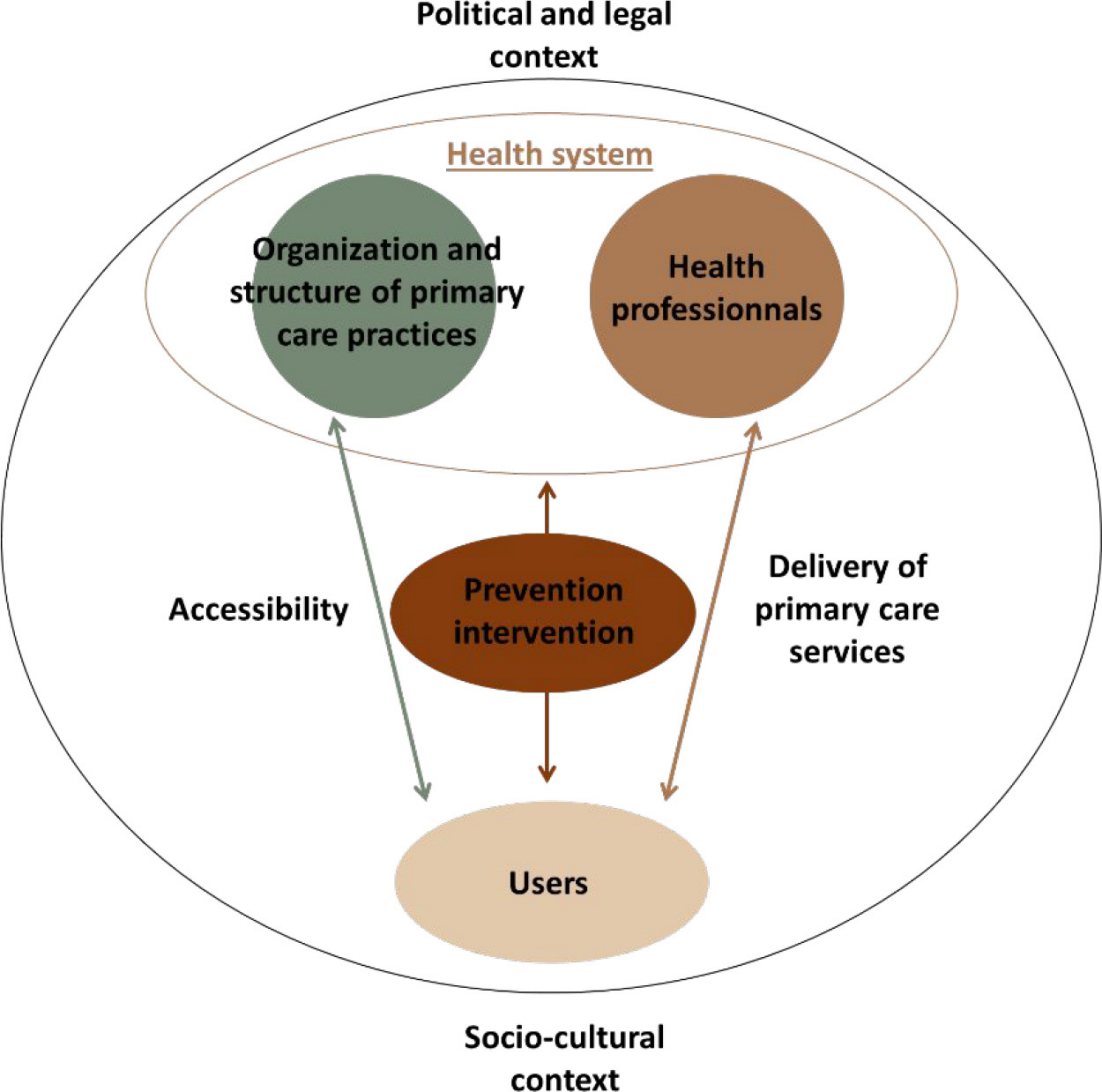
Analysis grid

The assembled data were then analysed using a thematic approach, coding the results in an analysis grid. This analysis grid (Figure 1) was designed using different sources. The first was the 'consolidated framework assessing PC organisation and performance' by Senn *et al*.^21^ This is a framework describing the organisation of PC from a very global point of view integrating dynamic interactions. With this objective in mind, it was adapted to prevention, one of the themes of PC. Thus, three contextual factors (health system, socio-cultural context, and political and legal context), two domains (delivery of PC services, and organisation and structure of PC practices) and one connecting construct (accessibility) were enlisted from this framework for the analysis grid. To this, three additional domains were added: users, health professionals (HPs), and preventive intervention. They were added inductively based on the data obtained in the overview of reviews that were included. This grid includes aspects related to the intervention, actors, functioning, and context of prevention in PC.

## Results

### Search results

**Figure 2.**
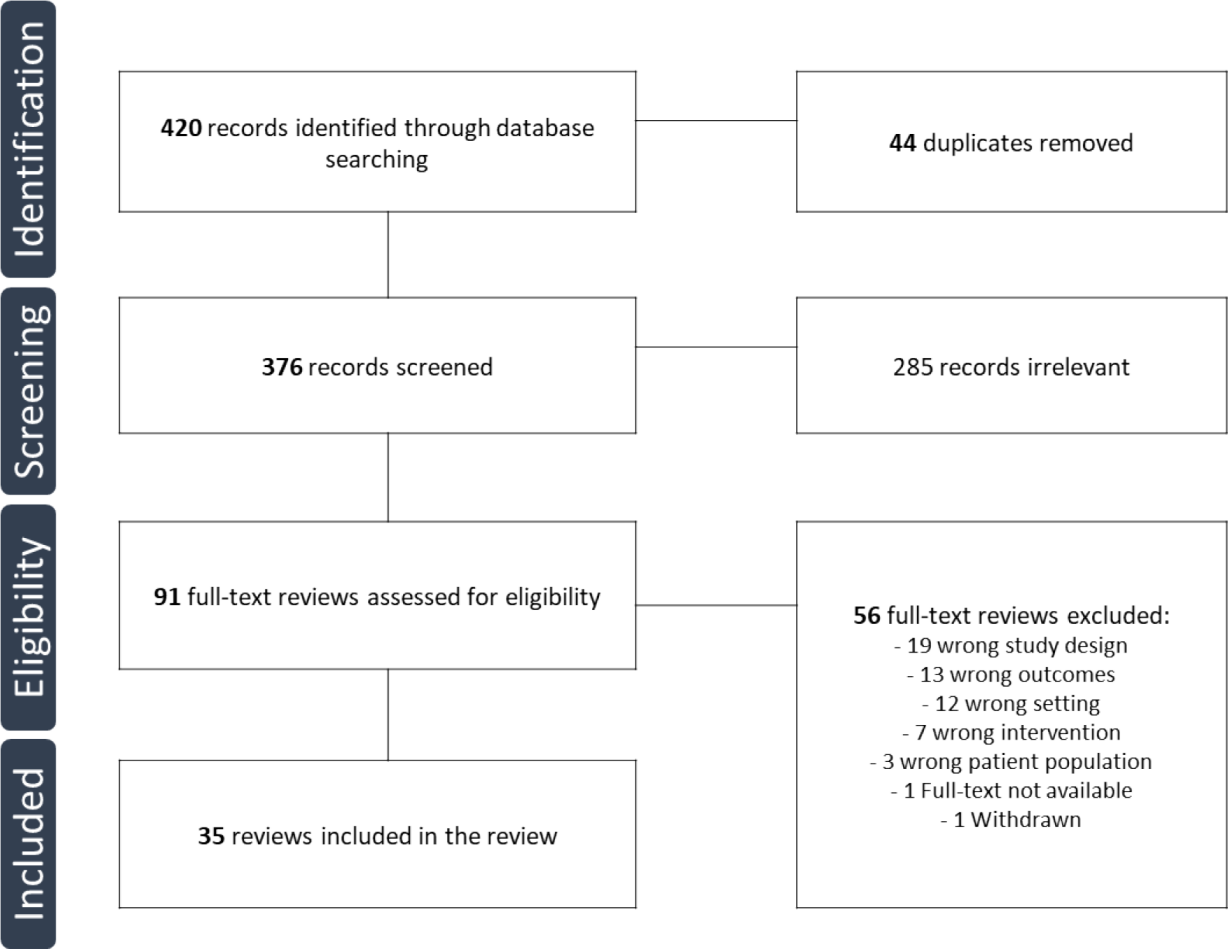
Flow diagram.

The search strategy allowed us to identify 420 records, of which 44 were duplicates. In total, 376 records were thus examined of which 285 were identified as irrelevant. Ninety-one full texts were assessed for eligibility, after which 35 articles were included (Figure 2).

### Characteristics of included reviews

The prevention topics are various, some reviews deal with a specific theme and four review deal with prevention from a general point of view. The reviews included are described in Supplementary Table S2. The analysis methods of the included reviews are mostly qualitative (*n* = 33); two reviews have a mixed-analysis method (qualitative and quantitative). Following the risk of bias assessment, 22 reviews were classified as having a low risk of bias, three a high risk of bias, and 10 with an unclear risk of bias.

### Main findings

A large number of barriers and facilitators were identified. These factors are heterogeneous with regard to their source (the patient, the professional, or the health system) and their level of action (individual, organisational, or contextual). All the results and some examples are shown in Supplementary Table S3.

### Individual

#### Health professionals

This topic concerns all individual factors related to HPs working in PC. Lack of time^22–40^ is the most cited constraint (*n* = 19). Receiving appropriate and relevant prevention training^22,26,31,34,36,40,41^ is the factor positively associated with prevention practice cited in the most reviews (*n* = 7).

#### Users

The most frequently found barrier, which is related to users, is lack of education and knowledge on the part of patients on the subject of prevention (*n* = 4), while the facilitator most frequently cited (*n* = 4) is the support of family and friends in the patient’s entourage.^34,40,42,43^

### Organisational

#### Organisation and structure of PC practices

This topic concerns organisation and environmental characteristics that may influence PC. These are the material and human resources that a PC provider requires to develop prevention in the practice and the ways in which they would be organised.^21^

There are several elements related to the organisation and structure of PC that can influence the practice of prevention by HPs. For example, the most cited barrier (*n* = 5) is the lack of equipment available for professionals to develop prevention in their practices.^24,26,31,39,41^ The mobilisation of an information system is the most frequent facilitator mentioned in the literature (*n* = 3) to develop prevention in PC.^39,40,44^

#### Delivery of PC services

This is defined as the process by which HPs deliver PC services to patients and the population.^21^ Providing recommendations and advice to patients^25,30,41,45,46^ is the main facilitator in this area (*n* = 5). The fact that certain prevention themes are given less priority than other health problems^22,29,36^ is a considerable barrier to the development of prevention in PC.

#### Prevention intervention

This topic is defined by all of the factors that relate to a prevention intervention, that is, the development or the components of the intervention, the tools used for the intervention, or the implementation of the intervention. Lack of information materials for patients^36,40^ hinder the development of prevention in PC. On the contrary, a low-cost intervention,^24^ integrated into routine activities,^24,47^ in a systematic way,^41^ which is simple to implement^24^ and adapted to the needs of the patients as well as to the reality of the services,^24^ will favour the development of prevention in PC.

#### Accessibility

Accessibility is defined as the possibility of receiving care when and where it is needed.^21^ There are four types of accessibility: time accessibility,^25,31,34–37,40,41,48,49^ geographical accessibility,^41,43,44^ accessibility of providers,^33,40,41,44,50^ and financial accessibility.^22,32,39–41,44,47,49,51^

The two most common barriers found (*n* = 4) are the lack of time for patients^25,31,48,49^ and the lack of economic support for patients.^22,32,40,49^ Conversely, offering time slots outside office hours is the most frequent (*n* = 2) facilitator for accessibility in the development of prevention in PC.^41,48^

### Contextual

#### Socio-cultural context

This theme is defined by Senn *et al* as '*the social status, education levels, self-confidence, behavioural context, culture and tradition*'.^21^ There are two socio-cultural contexts, that of the patient and that of the HPs. Both have an impact on prevention in the PC system. The patient’s socio-cultural background is the most frequently cited (*n* =6).

#### Political and legal context

The political and legal context is defined by Senn *et al* as '*a country’s political system, its legislative and regulatory setting*'.^21^ The unclear definition of the role of professionals^28,38^ is an example of political barriers to the development of prevention in PC. The use of legislation in the context of behavioural change,^40^ as is the case for tobacco, and the institutional promotion of prevention campaigns and messages^34^ are favourable for the development of prevention in PC.

## Discussion

### Summary

The factors acting on the integration of prevention in PC are numerous and varied. They can be classified in eight themes (health professionals, users, organisation and structure of PC practices, delivery of PC services, prevention intervention, accessibility, socio-cultural context, and political and legal context) according to their area of action. These themes are related to the individual, organisational, and contextual level of the healthcare system (Supplementary Table S3).

### Strengths and limitations

The reviews included in this overview of reviews are heterogeneous in terms of their subject matter and the methods of analysis used, and some of the reviews were assessed as having a high risk of bias. Also, this overview of reviews does not allow us to conclude whether certain factors are specific to certain themes (for example, cancer screening), or to certain professionals in particular (for example, GPs or nurses), nor the extent to which they influence each other. However, the objective here was to have an overall view of the factors influencing the routine practice of prevention.

### Comparison with existing literature

The results show that the implementation of prevention in PC goes far beyond the fact that patients are not sufficiently informed and professionals are not sufficiently trained. There are clearly factors linked to changes in the behaviour of patients and professionals^15^ and in their experiences and their emotions, but many other dimensions of the PC system— its organisation, its accessibility, the context, and the interactions within it — must also be taken into account. The multiplicity of factors involved and the dynamic relationships between them resonates with similar findings pertaining to other types of changes in the PC setting.^52^ PC must therefore be considered as a set of elements of differing natures operating at various levels, as depicted by the three-level framework: microsystem (clinical level), mesosystem (organisational level), and macrosystem (health-system level).^53^

Some of the factors identified in the literature can be modified, while others cannot (for example, patients' physio-pathological status or patient’s social norms). The factors that can be modified are mainly related to the organisation in nature (for example, providing more training for HPs, improving coordination between HPs, making suitable infrastructure available, developing a shared information system, and so on). Accordingly, a study found that the way in which office practices are organised is a predictor of better performance in terms of prevention.^54^ Thus, the development of prevention in PC cannot be conceived independently of a solid underlying organisation.

Many countries have recently reorganised their PC systems. For example, in England, integrated care systems (ICS) were legally established on 1 July 2022. France and Canada have also seen the development of coordinated practice structures, the aim of which is to coordinate a multidisciplinary team of HPs in the same area around a common health project.^55–57^ In France, for example, these coordinated exercise structures have a mandatory prevention mission.^57^ These organisation changes may provide an opportunity to work on integrating prevention into HP practices.

### Implications for research and practice

If more prevention is to be integrated into PC, all levels of the healthcare system must be involved in developing prevention interventions. Given the number and diversity of factors identified in this overview of reviews, it is essential to consider several strategies.

One of the major conditions for the development of prevention is the development of healthcare organisations. It would appear that these structures could remove barriers to the coordination and accessibility of HPs. A study shows that joint management of patients in primary care by several healthcare professionals improves the quality of care and reduces organisational constraints.^58^ Thus, it is not necessary to create a specific prevention system, but rather to reflect on how the PC system could take into account all the dimensions of prevention.

In conclusion, multiple factors influence the integration of prevention practices within PC, operating at distinct levels: the individual, organisational, and health-system levels. The organisation aspect of these factors is noteworthy and integrating the practice of prevention within existing healthcare organisations seems to be a way of removing certain barriers.

## Data

The dataset relied on in this article is available from the corresponding author on reasonable request.
